# Supplementary material for: Modeling glioblastoma heterogeneity as a dynamic network of cell states
Source: Mol Syst Biol. 2021 Sep 16;17(9):e10105. doi: 10.15252/msb.202010105 (PMC8444284; doi:10.15252/msb.202010105)
Supplement: Supplementary file 5 — Source Data for Figure 3 [file MSB-17-e10105-s001.zip › Figure3A_sourcedata/GSEA_3065/nosigdb_state4.GseaPreranked.1622718430994/ALLEN_113_ASTROL1FGFR3FOS.html]

Details for gene set ALLEN\_113\_ASTROL1FGFR3FOS[GSEA]

|  || Dataset | state4 |
| Phenotype | NoPhenotypeAvailable |
| Upregulated in class | na\_pos |
| GeneSet | ALLEN\_113\_ASTROL1FGFR3FOS |
| Enrichment Score (ES) | 0.6803382 |
| Normalized Enrichment Score (NES) | 2.1613872 |
| Nominal p-value | 0.0 |
| FDR q-value | 2.1249999E-4 |
| FWER p-Value | 0.001 |
Table: GSEA Results Summary

  

Fig 1: Enrichment plot: ALLEN\_113\_ASTROL1FGFR3FOS      
 Profile of the Running ES Score & Positions of GeneSet Members on the Rank Ordered List

  

| PROBE | GENE SYMBOL | GENE\_TITLE | RANK IN GENE LIST | RANK METRIC SCORE | RUNNING ES | CORE ENRICHMENT || 1 | GFAP |  |  | 0 | 0.995 | 0.2378 | Yes |
| 2 | S100B |  |  | 6 | 0.630 | 0.3876 | Yes |
| 3 | CRYAB |  |  | 10 | 0.494 | 0.5052 | Yes |
| 4 | NEAT1 |  |  | 41 | 0.315 | 0.5756 | Yes |
| 5 | ID2 |  |  | 56 | 0.292 | 0.6432 | Yes |
| 6 | NKAIN3 |  |  | 154 | 0.220 | 0.6803 | Yes |
| 7 | ZFP36L1 |  |  | 497 | 0.137 | 0.6583 | No |
| 8 | CST3 |  |  | 792 | 0.104 | 0.6361 | No |
| 9 | GLIS3 |  |  | 1032 | 0.086 | 0.6183 | No |
| 10 | SLC1A3 |  |  | 1069 | 0.083 | 0.6324 | No |
| 11 | SLC4A4 |  |  | 1160 | 0.078 | 0.6367 | No |
| 12 | KANK1 |  |  | 1395 | 0.067 | 0.6150 | No |
| 13 | NCKAP5 |  |  | 2563 | 0.034 | 0.4360 | No |
| 14 | BMPR1B |  |  | 2711 | 0.030 | 0.4197 | No |
| 15 | FGFR3 |  |  | 2779 | 0.029 | 0.4159 | No |
| 16 | FBXL7 |  |  | 3069 | 0.024 | 0.3752 | No |
| 17 | LRIG1 |  |  | 3101 | 0.023 | 0.3758 | No |
| 18 | DBI |  |  | 4122 | -0.017 | 0.2164 | No |
| 19 | ID4 |  |  | 4329 | -0.031 | 0.1908 | No |
| 20 | ADGRV1 |  |  | 4457 | -0.041 | 0.1802 | No |
| 21 | MAML2 |  |  | 4512 | -0.045 | 0.1823 | No |
| 22 | ADGRA3 |  |  | 4961 | -0.080 | 0.1295 | No |
| 23 | MEIS2 |  |  | 5012 | -0.084 | 0.1414 | No |
| 24 | PON2 |  |  | 5246 | -0.105 | 0.1292 | No |
| 25 | SLC7A11 |  |  | 5582 | -0.141 | 0.1092 | No |
Table: GSEA details [plain text format]

  

Fig 2: ALLEN\_113\_ASTROL1FGFR3FOS: Random ES distribution      
 Gene set null distribution of ES for **ALLEN\_113\_ASTROL1FGFR3FOS**
